# Pork as a Source of Omega-3 (*n*-3) Fatty Acids

**DOI:** 10.3390/jcm4121956

**Published:** 2015-12-16

**Authors:** Michael E.R. Dugan, Payam Vahmani, Tyler D. Turner, Cletos Mapiye, Manuel Juárez, Nuria Prieto, Angela D. Beaulieu, Ruurd T. Zijlstra, John F. Patience, Jennifer L. Aalhus

**Affiliations:** 1 Agriculture and Agri-Food Canada, Lacombe Research Centre, Lacombe T4L 1W1, AB, Canada; payam.vahmani@agr.gc.ca (P.V.); manuel.juarez@agr.gc.ca (M.J.); nuria.prieto@agr.gc.ca (N.P.); jennifer.aalhus@agr.gc.ca (J.L.A.); 2 Josera GmbH & Co. KG, Kleinheubach 63924, Germany; t.turner@josera.de; 3 Department of Animal Sciences, Stellenbosch University, Stellenbosch 7602, South Africa; cmapiye@sun.ac.za; 4 Prairie Swine Centre, Inc., Saskatoon S7H 3J8, SK, Canada; denise.beaulieu@usask.ca; 5 Department of Agricultural, Food and Nutritional Sciences, University of Alberta, Edmonton T6G 2R3, AB, Canada; ruurd.zijlstra@ualberta.ca; 6 Department of Animal Science, Iowa State University, Ames, IA 50011-3150, USA; jfp@iastate.edu

**Keywords:** pig, pork, *n*-3, omega-3, LNA, ETA, EPA, DHA

## Abstract

Pork is the most widely eaten meat in the world, but typical feeding practices give it a high omega-6 (*n*-6) to omega-3 (*n*-3) fatty acid ratio and make it a poor source of *n*-3 fatty acids. Feeding pigs *n*-3 fatty acids can increase their contents in pork, and in countries where label claims are permitted, claims can be met with limited feeding of *n*-3 fatty acid enrich feedstuffs, provided contributions of both fat and muscle are included in pork servings. Pork enriched with *n*-3 fatty acids is, however, not widely available. Producing and marketing *n*-3 fatty acid enriched pork requires regulatory approval, development costs, quality control costs, may increase production costs, and enriched pork has to be tracked to retail and sold for a premium. Mandatory labelling of the *n*-6/*n*-3 ratio and the *n*-3 fatty acid content of pork may help drive production of *n*-3 fatty acid enriched pork, and open the door to population-based disease prevention polices (*i.e.*, food tax to provide incentives to improve production practices). A shift from the status-quo, however, will require stronger signals along the value chain indicating production of *n*-3 fatty acid enriched pork is an industry priority.

## 1. Introduction

Over the last century, changes in agricultural production and patterns of food consumption have led to an increase in the omega-6 (*n*-6) to omega-3 (*n*-3) fatty acid ratio in human diets. The imbalance in the *n*-6 to *n*-3 ratio has been associated with numerous diseases, from cardiovascular and inflammatory diseases to diabetes and autoimmune disorders, and led to calls for rebalancing the ratio in the food supply [1]. Pork is the most widely eaten meat world-wide, accounting for 38% of meat production and over 36% of meat intake in the world [2]. Enriching pork among other meats represents a viable means to increase *n*-3 fatty acid consumption in humans, while leading to concomitant reductions in fatty acids associated with adverse health outcomes (e.g., saturated fatty acids (SFA)). This may be of particular importance in populations that do not consume fish or marine products, where red meat may contribute up to 30% of dietary long chain *n*-3 fatty acids [3]. The objectives of the present review are to examine where pork fits in the human diet, the fatty acid composition of pork, efforts to enrich pork with *n*-3 fatty acids when feeding flaxseed, and examination of practical barriers and possible strategies to help drive *n*-3 pork development and entry into the food supply.

## 2. Pork in Human Diets

**Figure 1:**
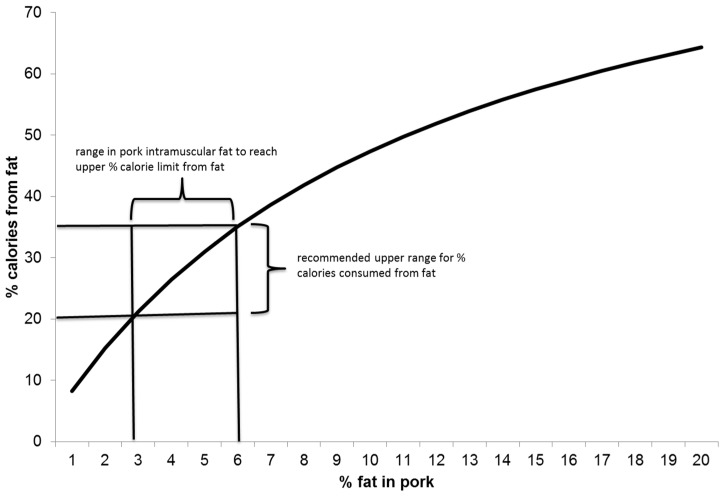
Calories in pork fat relative to recommendations for fat consumption.

The apparent link between plasma cholesterol and cardiovascular disease, and its association with SFA intake, led to recommendations to reduce the intake of SFA rich foods including red meat [4]. In response, swine industries in several countries adopted feeding and breeding strategies to reduce the fat content of pork. Results of these efforts were dramatic, leading to as little as 0.8%–1% intramuscular (marbling) fat, and increasing to a minimum of 1.5% marbling fat has since been recommended to ensure palatability [5]. Even at 2% marbling fat, lean pork contains 120–130 calories per 100 g serving, and only 15% of the calories are from fat. It would take pork marbling levels of 3%–6% to fall into the maximum recommended range of fat intakes for humans (20%–35% of dietary energy, Figure 1) [6]. In addition, if retail pork contains 2% intramuscular fat, the fat typically contains ~35%–40% SFA, equaling 5%–6% of total energy, which is again less than the maximum recommendation of 10% [6]. In light of these facts, and results of a meta-analyses of observational and epidemiological studies indicating SFA intake is not associated with cardiovascular disease risk [7,8], recommendations to substitute other protein sources for lean red meat in the human diet have been questioned [9,10]. On the other hand, even though the low fat and high protein contents of lean pork make it an excellent whole food for inclusion in human diets, complete pork carcasses contain ~47% fat [11], and this fat enters the food supply in the form of sausages, bacon, processed meats, and as lard in baked goods.

Common indicators of the healthfulness of fatty acid profiles (polyunsaturated fatty acid (PUFA)/SFA, and *n*-6/*n*-3 ratios) indicate there may be existing opportunities for their rebalancing in pork. In a survey of retail pork in England, the PUFA/SFA ratio of lean pork was 0.58 [12], which is greater than the 0.4 minimum recommended by the UK Department of Health [13], but an *n*-6/*n*-3 ratio of 7.2:1 was found, exceeding the recommended ratio of 4:1 [13]. Swine diets used in commercial intensive production are typically grain based, with the type of grain fed dependent on local availability and economics. Swine in the US and Canada are typically fed diets based on corn or small grains (barley/wheat). Juárez *et al.* [14] fed pigs on a barley/wheat/soybean based diet for 11 weeks prior to market and found a *longissimus* muscle (*i.e*., the largest steak/chop muscle running the length of the spine) PUFA/SFA ratio of 0.32 and a *n*-6/*n*-3 ratio of 4.5:1. Romans *et al.* [15] finished pigs using a typical corn/soybean based diet and found a *longissimus* muscle PUFA/SFA ratio of 0.24 and an *n*-6/*n*-3 ratio of 14.3:1. Thus, although lean pork has a desirable nutrient profile with high protein and low fat, there may still be some health benefit to adjusting the fatty acid composition (*i.e.*, reducing the *n*-6/*n*-3 ratio and increasing the PUFA/SFA ratio).

## 3. Pork Fatty Acid Composition

Lean pork contains a fairly constant proportion of cell membrane phospholipids, which are relatively rich in PUFA [16]. Lean pork also contains variable amounts of neutral lipid, composed mainly of SFA-rich triacyglycerol. The fatty acid composition of pork can thus be improved by reducing its total fat content, but if the fat content is too low, it can lead to palatability issues [17]. The composition of pork can also be influenced by diet, and diet adjustments can be used to improve pork fatty acid profiles without reducing total fat content [18,19]. Opportunities to use diet to change the fatty acid composition of meat are species specific, with major differences found between monogastric and ruminant livestock [20]. Pigs are monogastrics and categorized as homolipoid organisms [21], meaning their fatty acid composition closely reflects the fatty acid composition of their diet. In contrast to ruminant animals, in pigs, fatty acids are not metabolized to any great extent by microbes in the digestive tract prior to lipid digestion and absorption [20]. This makes pork a good candidate for enrichment with *n*-3 fatty acids.

**Figure 2:**
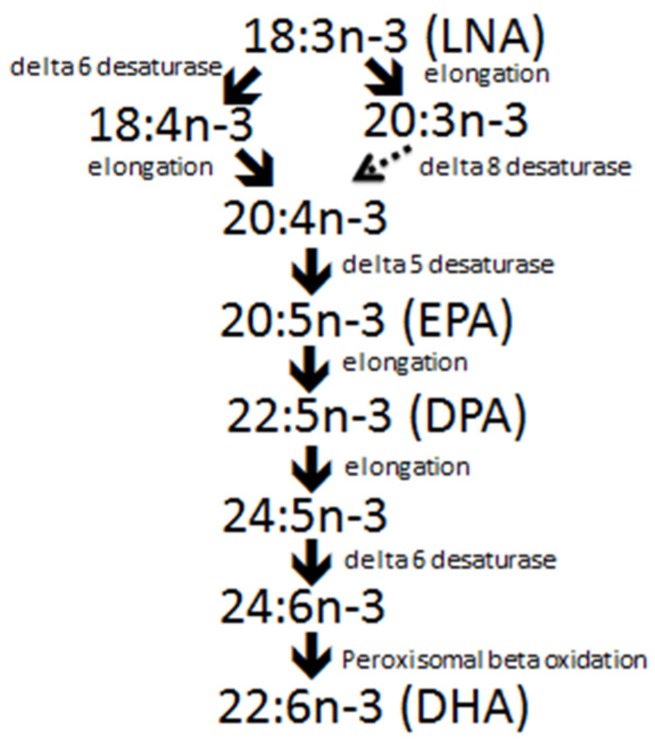
Pathways for *n*-3 fatty acid synthesis. LNA, alpha-linolenic acid; EPA, eicosapentaenoic acid; DPA, docosapentaenoic acid; DHA, docosahexaenoic acid.

Contrary to popular assumption, fat in red meats, including pork, is not solely composed of SFA (Table 1). When Juárez *et al.* [14] and Turner *et al.* [22] fed pigs a barley/wheat/soybean meal based diet, the *longissimus* muscle (denuded of epimysium and closely associated adipose tissue) was found to contain 2.9% total fat, and the fat contained 39% SFA, 47% monounsaturated fatty acids (MUFA), 11.5% PUFA, and the PUFA were comprised of 9.4% *n*-6 and 2.0% *n*-3 fatty acids. Within the *n*-3 fatty acids, α-linolenic acid (LNA, C18:3*n*-3) was the most concentrated at 22.7 mg/100 g of fresh tissue, and the most abundant long chain (LC) *n*-3 fatty acid was docosapentaenoic acid (DPA, C22:5*n*-3) at 11 mg/100g of fresh tissue. The LC *n*-3 fatty acids most widely studied for their health promoting properties include eicosapentaenoic acid (EPA, C20:5n-3) and docosahexaenoic acid (DHA, C22:6*n*-3) [23,24], and are concentrated in oily fish and marine products (e.g., microalgae). On the other hand, DPA is an intermediate in the pathway during DHA synthesis from EPA (Figure 2), and it is the most abundant LC *n*-3 fatty acid in meat and adipose tissue of terrestrial animals [25]. Docosapentaenoic acid which is freely converted between EPA and DHA, may have beneficial health effects on its own [26]. Consequently, when considering *n*-3 fatty acid nutrition, particularly in populations where oily fish or other LC *n*-3 fatty acid enriched marine products are not consumed, contributions of DPA made by terrestrial animals should be taken into consideration [27].

**Table 1:** Typical fatty acid composition of *longissimus* muscle and associated tissues in pork from pigs fed a barley/wheat/soybean meal diet.

Fatty Acid	mg/100 g Tissue					% of Fatty Acids				
	LM	AM + E	AM + E + SF	AM +E + SF + SCF	SEM	LM	AM + E	AM + E + SF	AM + E + SF + SCF	SEM
C16:0	718 ^d^	1523 ^c^	3033 ^b^	5782 ^a^	227	24.3	24.8	25.6	25.6	0.5
C18:0	378 ^d^	822 ^c^	1740 ^b^	3438 ^a^	128	12.8 ^c^	13.3 ^bc^	14.6 ^ab^	15.2 ^a^	0.5
ΣSFA	1147 ^d^	2458 ^c^	5001 ^b^	9659 ^a^	371	38.8 ^b^	39.9 ^ab^	42.2 ^a^	42.6 ^a^	0.9
C16:1-9c	101 ^d^	191 ^c^	313 ^b^	519 ^a^	25.8	3.39 ^a^	3.09 ^a^	2.66 ^b^	2.29 ^c^	0.12
C18:1-9c	1148 ^d^	2430 ^c^	4627 ^b^	9006 ^a^	357	38.6	39.4	39.2	39.9	0.8
C18:1-11c	116 ^d^	295 ^c^	420 ^b^	667 ^a^	24.0	3.96 ^b^	4.82 ^a^	3.59 ^c^	2.97 ^d^	0.06
ΣMUFA	1409 ^d^	3017 ^c^	5610 ^b^	10652 ^a^	415	47.4	49.0	47.5	47.2	0.9
C18:2*n*-6	189 ^d^	422 ^c^	822 ^b^	1656 ^a^	51.4	7.07	7.00	7.09	7.39	0.47
C18:3*n*-6	8.08 ^c^	13.8 ^c^	29.8 ^b^	66.5 ^a^	2.55	0.284 ^ab^	0.220 ^c^	0.258 ^bc^	0.295 ^a^	0.012
C20:2*n*-6	5.54 ^d^	15.5 ^c^	37.2 ^b^	85.9 ^a^	2.28	0.205 ^d^	0.262 ^c^	0.322 ^b^	0.385 ^a^	0.019
C20:3*n*-6	6.92 ^d^	10.1 ^c^	13.4 ^b^	21.5 ^a^	0.761	0.263 ^a^	0.166 ^b^	0.115 ^bc^	0.095 ^c^	0.020
C20:4*n*-6	46.0 ^d^	59.7 ^c^	65.8 ^b^	75.4 ^a^	2.03	1.77 ^a^	1.00 ^b^	0.580 ^c^	0.342 ^c^	0.120
C22:4*n*-6	1.35 ^d^	8.66 ^c^	11.8 ^b^	17.1 ^a^	0.59	0.048 ^d^	0.145 ^a^	0.102 ^b^	0.076 ^c^	0.005
Σ*n*-6	249 ^d^	516 ^c^	950 ^b^	1856 ^a^	55.0	9.36	8.57	8.21	8.29	0.61
C18:3*n*-3	22.7 ^c^	41.3 ^c^	89.5 ^b^	186 ^a^	8.22	0.818	0.669	0.758	0.824	0.067
C20:3*n*-3	3.01 ^c^	5.75 ^c^	15.6 ^b^	36.7 ^a^	1.22	0.104 ^c^	0.092 ^c^	0.131 ^b^	0.162 ^a^	0.008
C20:5*n*-3	6.35	5.26	5.72	7.87	0.68	0.235 ^a^	0.0854 ^b^	0.0484 ^bc^	0.0349 ^c^	0.016
C22:3*n*-3	5.40 ^a^	0.918 ^c^	1.79 ^bc^	2.52 ^b^	0.50	0.204 ^a^	0.0134 ^b^	0.0141 ^b^	0.0108 ^b^	0.011
C22:5*n*-3	11.0 ^d^	15.4 ^c^	20.8 ^b^	30.4 ^a^	1.1	0.422 ^a^	0.257 ^b^	0.181 ^bc^	0.137 ^c^	0.029
C22:6*n*-3	5.45 ^c^	6.42 ^bc^	8.71 ^b^	12.7 ^a^	0.88	0.209 ^a^	0.109 ^b^	0.0773 ^b^	0.0581 ^b^	0.024
Σ*n*-3	54.0 ^c^	75.1 ^c^	142 ^b^	276 ^a^	10.8	1.99 ^a^	1.22 ^b^	1.21 ^b^	1.22 ^b^	0.13
ΣPUFA	306 ^d^	596 ^c^	1104 ^b^	2158 ^a^	66	11.5	9.88	9.52	9.63	0.7
TOTAL	2922 ^d^	6140 ^c^	11,795 ^b^	22,577 ^a^	821	100	100	100	100	0
*n*-6/*n*-3	4.77 ^b^	7.00 ^a^	6.81 ^a^	6.76 ^a^	0.26					
PUFA/SFA	0.301	0.250	0.228	0.228	0.025					

LM, *longissimu*s muscle; AM + E, all muscles in loin + epimysium; AM + E + SF, AM + E + seam fat; AM + E + SF + SCF, AM + E + S + subcutaneous fat; SEM, standard error of the mean. SFA, saturated fatty acids; MUFA, monounsaturated fatty acids; PUFA, polyunsaturated fatty acids. ^a,b,c^ For mg/100 g and % data, means within a row with different superscripts are significantly different at *p* < 0.05.

The fatty acid composition of pig *longissimus* is the muscle most reported in the literature. Scientifically it is expedient to report fatty acid compositions on pure muscle, but when examining servings of pork, it is important to consider contributions made by various tissues. When Juárez *et al.* [14] and Turner *et al.* [22] combined *longissimus* and other muscles in the primal loin cut, including epimysium and closely associated adipose tissues, the fat content was 6.1% (*i.e.*, increased from 2.9% fat in pure *longissimus* muscle). When intermuscular (seam) fat was further included, the fat content of pork increased to 11.8%, and when 5 mm of overlying backfat was added to complete a commercially trimmed pork chop, the total fat content increased to 22.6%. The addition of fatty tissues to pure muscle also increased the percentage of SFA in total fat, and increased the *n*-6/*n*-3 ratio. In addition, when fatty tissues were included with pure muscle, LNA remained as the most abundant *n*-3 fatty acid, but the second most abundant *n*-3 fatty acid changed from DPA to eicosatrienoic acid (ETA, C20:3*n*-3). Despite the reduction in the %PUFA in the fat because the total fat increased, the actual amount of PUFA on a mg/100 g of fresh tissue basis increased from 306 mg to 2158 mg, and the *n*-3 fatty acids increase from 54 to 276 mg per 100g. Knowing the content of *n*-3 fatty acids in various cuts of pork is therefore of considerable importance as these help define the pig feeding practices required to meet label (enrichment) claims for *n*-3 fatty acids. For example, in Canada to make a retail label claim for *n*-3 fatty acid enrichment, a serving of food must contain at least 300 mg *n*-3 fatty acids [28]. If the serving only contains *longissimus* muscle, the *n*-3 fatty acid content would need to be increased 5–6 fold to reach enrichment status. On the other hand, a commercial loin chop (all muscles, seam and 5 mm of back fat) would likely need limited *n*-3 fatty acid supplementation in pig diets to reach 300 mg *n*-3 fatty acids per serving. Similarly, other higher fat cuts such as back or side ribs and bacon could also meet this label claim with limited supplementation.

**Figure 3:**
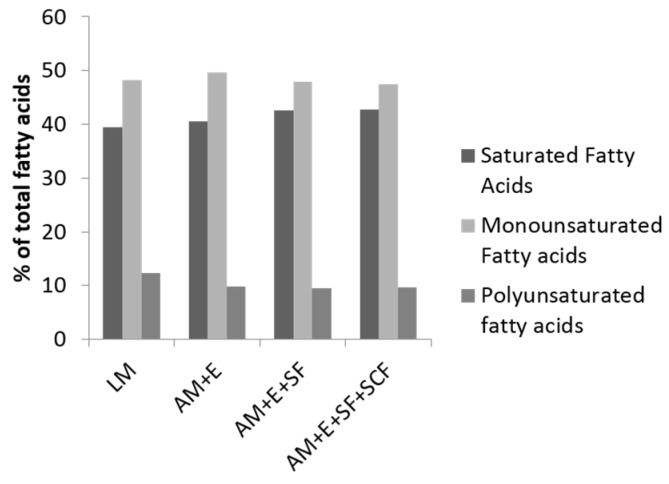
The percentage of saturated fatty acid (SFA), monounsaturated fatty acid (MUFA), and polyunsaturated fatty acid (PUFA) in total fatty acids in cuts of pork loin (LM, *longissimus* muscle; AM+E all loin muscles + epimysium; AM + E + seam fat; AM + E + SF + subcutaneous fat).

As previously mentioned, reducing the marbling fat content of lean pork improves the fatty acid composition by increasing the proportion of PUFA and the PUFA/SFA ratio. If extra-muscular fatty tissues are then added to pork cuts, the proportion of SFA goes up (Table 1). As illustrated in Figure 3, however, when extra-muscular sources of fatty tissue are included in pork cuts, changes in fatty acid composition are not striking. This is because, even when as little as 2.9% intramuscular fat is present, the content of SFA rich triacylglycerols are still enough to overwhelm the contribution made by PUFA rich phospholipids. In practical terms, the fatty acid profile of pork with enough intramuscular fat to ensure palatability is not very different from higher fat products such as pork sausage. The difference in healthfulness is more related to the total fat content rather than the fatty acid profile. It is also worth re-emphasizing that the most abundant class of fatty acids in pork are MUFA, and these are the main fatty acids in the heart healthy Mediterranean diet [29]. Moreover, approximately ~25% of the SFA in pork is stearic acid (C18:0), which is noted to have neutral effects on plasma cholesterol [30].

## 4. Enriching Pork with *n*-3 Fatty Acids

### 4.1. Initial Efforts to Improve Pork Fatty Acid Composition

The idea of modifying the fatty acid composition of pork to make it more healthful is not new. To improve the healthfulness of pork fatty acid profiles, Koch *et al.* [31] fed pigs safflower oil and achieved large increases in pork PUFA in the form of linoleic acid (LA, C18:2*n*-6). Stewart *et al.* [32] also fed a diet rich in LA, and achieved increased PUFA levels in pork and lard. When the LA enriched pork and foods made with LA enriched lard were fed to young women (aged 19–24) they significantly lowered total plasma and low-density lipoprotein (LDL) cholesterol, and increased PUFA and reduced SFA and MUFA in plasma lipids and erythrocytes. The pig diet, however, had *n*-6/*n*-3 fatty acid ratios of ~10:1, and with today’s understanding that lower ratios may impart even further benefits, there have been a number of studies conducted attempting to increase pork PUFA by increasing the *n*-3 fatty acid content.

### 4.2. Efforts to Increase n-3 Fatty Acids in Pork

The ability to substantially alter the *n*-3 fatty acid content of pork was demonstrated in 1972 by Anderson *et al.* [33] when studying LNA turnover. Feeding 20% flaxseed oil for two months to six month old pigs increased fat depot concentrations of LNA from 1% to 15%. Given the understanding that increasing the *n*-3 fatty acid content of pork may benefit consumers, and that feeding flaxseed instead of extracted flaxseed oil may reduce input costs, Cunnane *et al.* [34] fed 5% ground flaxseed to weaned pigs for 8 weeks and found several fold increases in LNA and its elongation and desaturation products in a number of tissues. Since this time, a number of studies have been conducted feeding flaxseed, flaxseed oil or sources of LC *n*-3 fatty acids (*i.e.*, fish meal, fish oil, and marine algae) and results have been extensively reviewed [16,18,19,35,36]. Cherian *et al.* [37], Romans *et al.* [15], Riley *et al.* [38] and Ahn *et al.* [39] conducted intensive studies on the effects of feeding 0 to ~15% flaxseed on pork quality and fatty acid composition, Romans *et al.* [40] and Juárez *et al.* [41] investigated feeding flaxseed for different durations, and Fontanillas *et al.* [42] and Huang *et al.* [43] investigated the evolution of *n*-3 fatty acids in pig tissues over the feeding period. Maximum levels of *n*-3 fatty acid deposition were comparable with enrichments achieved by Anderson *et al.* [33] when feeding flaxseed oil. To date, most studies feeding higher levels of flaxseed or flaxseed oil have found increased tissue contents of LNA, EPA and DPA, but not DHA. For example, Martínez-Ramírez *et al.* [44] found no increase in DHA when feeding pigs diets containing 7%–10% flaxseed, but interestingly, the amounts of other LC *n*-3 fatty acids deposited in pork were independent of whether the flaxseed was fed early or late in the feeding period. When feeding a limited amount of flaxseed (~2%), however, Enser *et al.* [45] found a reduction in the *n*-6/*n*-3 ratio from 9:1 to 5:1 and an increase in DHA. The pathway for *n*-3 fatty acid synthesis from LNA to DHA requires delta-6 desaturation in two places (Figure 2), and the increase in DHA was attributed to the low level of flaxseed supplementation. This means enough LNA was supplied to provide substrate for delta-6 desaturation to C18:4*n*-3, but not enough to out complete for delta-6 desaturase activity later in the pathway (*i.e.*, conversion of C24:5*n*-3 to C24:6*n*-3). Overall, feeding sources of *n*-3 fatty acids to pigs increased their content in pork, but results have been variable, and the differences attributed to the source, amount and type of *n*-3 fatty acids fed, the duration of feeding, the type of feed processing, the weight or age of pigs fed and their gender. Nevertheless, Nguyen *et al.* [46] found the mathematical relationship between the amount of LNA fed and deposited in pork was strong within a study (*R*^2^ = 0.98), but lower when data from several studies are incorporated into a regression (*R*^2^ = 0.68). Consequently, producers seeking to develop a strategy to produce *n*-3 enriched pork will likely be able to achieve consistency, but a standardized feeding program will be required based on in-house development rather than solely on strategies reported in the literature.

### 4.3. An Example of n-3 Enriched Pork and Post-Production Considerations

Results from Juárez *et al.* [14] and Turner *et al.* [22] provide an example of *n*-3 fatty acid enrichments in pork that might be attained when feeding optimally processed flaxseed to pigs, and factors that need to be considered when developing feeding strategies to meet *n*-3 fatty acid enrichment goals (Table 2). Pigs were fed a diet containing 10% flaxseed co-extruded 50:50 with field peas to optimize LNA digestibility [47]. The diet was fed from 48 to 121 kg body weight over an 11 week period resulting in increased LNA and total *n*-3 fatty acids from 0.82 and 1.99% of total *longissimus* muscle fatty acids to 5.76 and 8.94% respectively. This translated into an increase of LNA and total *n*-3 fatty acids from 22.7 and 54.0 mg per 100 g serving of pork to 145 and 217 mg respectively. These amounts of *n*-3 fatty acids would, however, not qualify for a source claim in a number of countries including Canada and the European Union, but may have potential in the United States. As mentioned previously, to be labelled as a source of *n*-3 fatty acids in Canada, a serving of food serving has to have 300 mg of total *n*-3 fatty acids [28]. In the USA, food servings with ≥160 mg or ≥320 mg LNA can be referred to as a “source” or “rich” in LNA respectively, and claims cannot be made for EPA and DHA [48]. In the European Union, foods with 300 mg LNA or 40 mg combined EPA and DHA per serving can be labeled as a source of *n*-3 fatty acids, and foods with 600 mg LNA or 80 mg combined EPA and DHA can be labeled as rich in *n*-3 fatty acids [49]. Source claims in all countries would, however, be possible when all muscles and fatty tissues were included in a retail pork chop. In fact, the amount of *n*-3 fatty acids in commercially trimmed pork chops was 10 times more than required for a source claim in Canada (3360 mg per 100 g serving) [22]. A source claim for combined EPA and DHA could also be made in the European Union (71.5 mg), but could only be considered a rich source if DPA was included in LC *n*-3 fatty acids (174 mg). The ability to make a source claim is, therefore, dependent on the country, what tissues are included in a serving of pork, the type of cut and the *n*-3 fatty acids considered to be LC. Consideration should, however, also be given to what consumers actually eat, as often some external fat may be trimmed before consumption. Therefore, ensuring consistent *n*-3 fatty acid enrichment and consumption might be most easily attained through development of further processed *n*-3 enriched pork products (e.g., sausages) and secondary products prepared with enriched lard (e.g., baked goods). Notably, when fatty tissues are added to pure muscle, the second most abundant *n*-3 fatty acid in pork changes from DPA to ETA (up to 381 mg per 100 g serving), and ETA has been shown to have a photo-protective effect in human skin [50]. There is also some limited evidence for delta-8 desaturase activity [51], converting ETA to C20:4*n*-3 in liver, which may gain importance in LC *n*-3 fatty acid synthesis as ETA concentrations increase (Figure 2).

**Table 2:** The fatty acid composition of *longissimus* muscle and associated tissues in pork from pigs fed a diet supplemented with 10% flaxseed.

Fatty Acid	mg/100 g Tissue					% of Fatty Acids				
	LM	AM + E	AM + E + SF	AM + E + SF + SCF	SEM	LM	AM + E	AM + E + SF	AM + E + SF + SCF	SEM
C16:0	571 ^d^	1180 ^c^	2227 ^b^	4169 ^a^	152	22.1 ^a^	20.1 ^b^	20.5 ^b^	19.8 ^b^	0.3
C18:0	318 ^d^	678 ^c^	1326 ^b^	2545 ^a^	102	12.4	11.7	12.2	12.1	0.3
ΣSFA	931 ^d^	1953 ^c^	3731 ^b^	7040 ^a^	265	36.0 ^a^	33.4 ^b^	34.3 ^b^	33.5 ^b^	0.6
C16:1-9c	66.6 ^d^	113 ^c^	177 ^b^	295 ^a^	12.0	2.59 ^a^	1.94 ^b^	1.68 ^bc^	1.41 ^c^	0.10
C18:1-9c	873 ^d^	1871 ^c^	3380 ^b^	6727 ^a^	218	33.8 ^a^	32.2 ^b^	31.7 ^b^	32.2 ^b^	0.5
C18:1-11c	76.7 ^d^	196 ^c^	265 ^b^	419 ^a^	14.3	3.06 ^b^	3.39 ^a^	2.53 ^c^	2.01 ^d^	0.08
ΣMUFA	1045 ^d^	2259 ^c^	3991 ^b^	7724 ^a^	256	40.7 ^a^	38.9 ^ab^	37.4 ^bc^	36.9 ^c^	0.6
C18:2*n*-6	225 ^d^	652 ^c^	1207 ^b^	2470 ^a^	55.0	9.68 ^b^	11.4 ^a^	11.5 ^a^	11.9 ^a^	0.34
C18:3*n*-6	6.38 ^d^	12.8 ^c^	25.1 ^b^	53.0 ^a^	1.54	0.26	0.223	0.236	0.253	0.011
C20:2*n*-6	6.40 ^d^	24.7 ^c^	47.1 ^b^	106 ^a^	2.75	0.27 ^b^	0.437 ^a^	0.454 ^a^	0.509 ^a^	0.025
C20:3*n*-6	5.86 ^c^	7.83 ^bc^	10.0 ^b^	15.9 ^a^	0.79	0.26 ^a^	0.141 ^b^	0.099 ^c^	0.076 ^c^	0.012
C20:4*n*-6	28.2 ^c^	34.3 ^b^	36.6 ^b^	42.3 ^a^	1.9	1.35 ^a^	0.626 ^b^	0.372 ^c^	0.205 ^c^	0.06
C22:4*n*-6	1.23 ^c^	3.87 ^b^	5.27 ^b^	7.95 ^a^	0.56	0.05 ^ab^	0.067 ^a^	0.049 ^ab^	0.037 ^b^	0.007
Σ*n*-6	266 ^d^	723 ^c^	1306 ^b^	2643 ^a^	59	11.6	12.7	12.5	12.7	0.4
C18:3*n*-3	145 ^d^	614 ^c^	1290 ^b^	2800 ^a^	71	5.76 ^d^	10.6 ^c^	12.1 ^b^	13.4 ^a^	0.30
C20:3*n*-3	21.5 ^d^	82.2 ^c^	166 ^b^	381 ^a^	11.6	0.85 ^c^	1.42 ^b^	1.55 ^b^	1.82 ^a^	0.053
C20:5*n*-3	23.3 ^d^	31.9 ^c^	38.8 ^b^	55.3 ^a^	1.7	1.08 ^a^	0.573 ^b^	0.385 ^c^	0.268 ^c^	0.05
C22:3*n*-3	2.48 ^b^	2.27 ^b^	3.06 ^b^	4.75 ^a^	0.40	0.10 ^a^	0.038 ^b^	0.029 ^b^	0.023 ^b^	0.005
C22:5*n*-3	21.1 ^d^	39.5 ^c^	59.7 ^b^	102 ^a^	3.0	0.95 ^a^	0.705 ^b^	0.575 ^c^	0.491 ^c^	0.038
C22:6*n*-3	3.85 ^d^	8.13 ^c^	10.6 ^b^	16.2 ^a^	0.77	0.18 ^a^	0.146 ^b^	0.105 ^c^	0.079 ^c^	0.011
Σ*n*-3	217 ^d^	778 ^c^	1569 ^b^	3360 ^a^	85	8.94 ^d^	13.5 ^c^	14.7 ^b^	16.1 ^a^	0.39
ΣPUFA	486 ^d^	1506 ^c^	2883 ^b^	6018 ^a^	143	20.6 ^c^	26.3 ^b^	27.4 ^ab^	28.9 ^a^	0.7
TOTAL	2519 ^d^	5788 ^c^	10,683 ^b^	20,881 ^a^	627	100	100	100	100	0
*n*-6/*n*-3	1.28 ^a^	0.935 ^b^	0.850 ^c^	0.788 ^c^						
PUFA/SFA	0.581 ^b^	0.796 ^a^	0.807 ^a^	0.869 ^a^						

LM, *longissimus* muscle; AM+E, all muscles in loin + epimysium; AM + E + SF, AM + E + seam fat; AM + E + SF + SCF, AM + E + S + subcutaneous fat; SEM, standard error of the mean. SFA, saturated fatty acids; MUFA, monounsaturated fatty acids; PUFA, polyunsaturated fatty acids. ^a,b,c^ For mg/100 g and % data, means within a row with different superscripts are significantly different at *p* < 0.05.

Currently, production of pork enriched with *n*-3 fatty acids is possible, but it is not clear which *n*-3 fatty acid should be enriched, to what extent they should be enriched and in what tissues. Feeding pigs a limited amount of flaxseed can rebalance the *n*-6/*n*-3 ratio in pork [45], but a healthier *n*-6/*n*-3 ratio is not something that can be advertised or put on a label in most countries. Feeding increased amounts of flaxseed can yield pork that can be labelled as a source, or rich source of *n*-3 fatty acids, but if consumers trim visible fat or the fat is lost during cooking, purchased pork may differ from pork consumed. Even when pork is enriched with enough *n*-3 fatty acids to allow for a source claim, beneficial effects of consuming such pork have not been extensively investigated. Coats *et al.* [52] found regular consumption of pork enriched with LC *n*-3 fatty acids by feeding fish meal increased erythrocyte DHA by 15%, and compared to a control group, serum triacylglycerol decreased to a greater extent and thromboxane production increased to a lesser extent. Using a rabbit model, Vossen *et al.* [53] fed pork enriched with LNA or LNA plus LC *n*-3, and found only pork enriched with LNA plus LC *n*-3 fatty acids reduced the total plasma cholesterol to high-density lipoprotein cholesterol (HDL-C) ratio. Clearly, it would be of benefit to conduct additional clinical trials to establish the health effects of consuming commercial pork compared to pork enriched with *n*-3 fatty acids, and factors considered should include the *n*-6/*n*-3 ratio, the amount and composition of *n*-3 fatty acids and the overall fat content of the pork in different meat cuts.

## 5. Practical Barriers Limiting *n*-3 Pork Development and Entry into the Food Supply

### 5.1. The Call for n-3 Enriched Meat Unfulfilled to Date

Simopoulos [54] indicated that it is essential in the process of returning the *n*-3 fatty acids into the food supply, that the balance of *n*-6/*n*-3 fatty acids in the diet that existed during human evolution is maintained. To date fish-meal, flaxseed, and marine algae in poultry feeds have increased the *n*-3 fatty acid content of egg yolks and led to the supply of *n*-3 fatty acid-enriched eggs in the marketplace [55]. Simopoulos [54] noted research on the production of *n*-3 fatty acid-enriched products from poultry, beef, lamb, pork, milk and bakery products was ongoing. Taking advancements in the ability to enrich animal products with *n*-3 fatty acids into account, Givens and Gibbs [56] estimated potential dietary intakes of EPA+DHA from foods derived from animals fed enriched diets would be ~231 mg/day, which would double current intakes in the UK, and help meet the recommend intake of 450 mg/day. Currently, however, in most countries, despite the technological capability to do so, the availability of *n*-3 enriched meats, including pork, is not widespread.

### 5.2. Why Production of n-3 Enriched Pork Has Not Been Adopted

#### 5.2.1. Visibility

When the SFA content of red meat was associated with increased plasma cholesterol and cardiovascular disease in the 1960s, consumers could visually identify and select meats with lower fat contents, and animal producers selected and fed animals to meet consumer demands for leaner meat. In developed countries, sweeping changes in pork production took place within a value chain geared to produce commodity pork for mass markets. The cost of pork production and the retail price of pork were not increased due to changes in production strategies. Currently, even though the production of *n*-3 fatty acid enriched meats, including pork, has been encouraged, changing fat composition in retail pork has not been as successful as previous efforts to reduce the total fat content of pork. As opposed to the total fat content, the fatty acid composition of retail pork is not visible to consumers.

#### 5.2.2. Challenges along the Value Chain

There are several challenges to producing *n*-3 enriched pork along the value chain. Increasing the *n*-3 fatty acid content of pork can lead to increased input costs depending on feedstuffs available and requirements for processing. Entry into the *n*-3 pork market may also have lagged because feeding high levels of *n*-3 fatty acids can lead to fat softness and palatability problems [14]. Effects on freshly cooked pork chops have, however, been limited [14,39,57] and most negative effects have been found in cooked/reheated pork chops and freshly cooked ground pork with excessive *n*-3 fatty acid enrichments. Once *n*-3 fatty acid enriched pork is produced, it also requires vertical integration from production to retail, along with differentiated marketing and higher prices to cover input costs, distribution costs and profits for producers. Producing *n*-3 fatty acid enriched pork to meet source claims also requires regulatory approval for package labelling, defining what will be included in portions, what production strategies are needed to meet enrichment requirements, and also added costs for fatty acid analysis of feeds and pork during product development and for quality control. Strategies to drive an industry-wide shift towards *n*-3 fatty acid enriched pork must, therefore, be developed if a clear goal for producers is widespread production and marketing of *n*-3 enriched pork.

## 6. Strategies to Encourage Production and Market Availability of *n*-3 Fatty Acid Enriched Pork

Strategies to encourage production and market availability of *n*-3 fatty acid enriched pork will likely require concerted efforts along the value chain. Producer entry into the *n*-3 fatty acid enriched pork market may be enhanced with the understanding that only limited supplementation of *n*-3 fatty acids in diets is required to meet label claims when contributions of all tissues in a serving are included [22]. This also opens possibilities for feeding oils or oilseeds that may not be as highly enriched with LNA as flaxseed (e.g., whole canola or canola oil). An industry-wide shift in pork production practices might also be driven by mandatory labelling of *n*-3 fatty acids and the *n*-6/*n*-3 ratio in meat, making these visible to consumers. When consumers know the *n*-3 fatty acid and *n*-6/*n*-3 ratio in foods, it provides the opportunity to select more healthful foods, and impetus to the industry to find lower cost production strategies. In this way, pork may not have to reach specified amounts of *n*-3 fatty acids to meet regulatory approval as a source of *n*-3 fatty acids, but could contribute a greater quantity of *n*-3 fatty acids to the human diet, and at the very least, not further imbalance in the *n*-6/*n*-3 ratio. Analyzing the fatty acid composition of pork in the packing house or at retail by traditional means (*i.e.*, gas chromatography) would not be cost effective or practical, but newer non-invasive technologies including near infra-red reflectance spectrophotometry (NIRS), NIRS hyperspectral imaging, or Raman spectroscopy [58,59,60,61] may hold promise to deliver analyses in seconds *versus* days, and coupled to new tracking systems, such as radio frequency identifier tags, may be able to deliver this information to the consumer at retail. Mandatory labelling of *n*-3 fatty acids and the *n*-6/*n*-3 ratio might also open the door to population-based prevention policies (*i.e.*, food tax to drive nutritional improvements through changes in production practices), which could generate health gains while paying for themselves through future reductions of health-care expenditures [62], or by providing incentives to producers with specified amounts and types of *n*-3 fatty acids in their pig feed. Market pull could also be generated through inclusion of healthier sources of fatty acids (*i.e.*, *n*-3 enriched lard) in baked goods, and further processed products that already qualify for nutritional labels, and this might in turn result in healthier pork meat as a byproduct. For all the strategies, however, it is clear that there needs to be more defined incentives provided to producers, and a stronger signal that changes are required along the value chain.

## 7. Conclusions

Currently retail pork is not considered a source of *n*-3 fatty acids, and in fact suffers from an imbalanced *n*-6/*n*-3 fatty acid ratio related to modern feeding practices. Pork is the most consumed meat in the world, and its fatty acid content and composition is directly influenced by diet. There have been calls to correct the imbalanced *n*-6/*n*-3 ratio in foods, including pork, and although this correction would seem to be a simple fix by modifying pig diets, adoption of such practices is not widespread. Producing *n*-3 enriched pork may increase production costs, enriched pork has to be tracked to retail, and the pork must be sold at a premium to recover added costs and provide profit for the effort. Labelling pork as a source of *n*-3 fatty acids also requires regulatory approval, development costs and costs for quality control to maintain enrichment status. As a result, *n*-3 enriched pork will likely continue to command a limited market share, and only be available to those willing to pay a premium. Several strategies to drive an industry wide shift towards *n*-3 fatty acid enriched pork production are possible including mandatory labelling of the *n*-3 fatty acid content and *n*-6/*n*-3 ratio, and development of population based prevention polices. This would allow consumers to make choices based on valued attributes, and provide for natural market segmentation without having to reach specific amounts of *n*-3 fatty acids per serving. When coupled with improvements in the speed of non-invasive fatty acid analyses and tracking technologies, we could be on the verge of meeting health conscious consumers growing demand for nutritional information, while providing impetus to pork value chain to make producing pork with a higher *n*-3 fatty acid content and lower *n*-6/*n*-3 ratio an industry wide priority.
